# Why viewers engage in sports live streaming: the psychological pathways from SMI characteristics to participative and sharing behaviors

**DOI:** 10.3389/fpsyg.2026.1763064

**Published:** 2026-05-20

**Authors:** Yi Ding, Beili Tu, Ruonan Tu, Yi Peng

**Affiliations:** 1 School of Digital Economy and Trade, Wenzhou Polytechnic, Wenzhou, China; 2 Department of International Trade, Changwon National University, Changwon, Republic of Korea; 3 School of Physical Education, Putian University, Putian, Fujian, China

**Keywords:** emotional bond, flow experience, social media influencer (SMI) characteristics, sports live streaming, viewer engagement

## Abstract

**Introduction:**

Sports live streaming has become an important online setting in which social media influencers (SMIs) can shape how viewers respond to content. Previous studies have examined influencer-related traits and flow experience, but these factors have rarely been considered together in sports live streaming. To address this gap, the present study applies the Stimulus-Organism-Response (SOR) framework to examine whether flow experience mediates the relationship between influencer characteristics and viewer behavior, and whether emotional bond moderates this process.

**Methods:**

Data were collected through a cross-sectional survey of 617 Chinese viewers with prior experience watching sports live streams. Established scales were used to measure SMI expertise, credibility, attractiveness, flow experience, participative behavior, sharing behavior, and emotional bond. Structural equation modeling was then used to examine the proposed relationships.

**Results:**

The analysis shows that attractiveness had the strongest positive effect on flow experience, while expertise and credibility also showed significant positive effects. Flow experience was closely associated with both participative behavior and sharing behavior. Emotional bond further reinforced the positive effects of expertise and credibility on flow experience, although it did not significantly change the impact of attractiveness. Participative behavior and sharing behavior did not reflect a simple sequential progression of engagement. Instead, they functioned more like two relatively independent forms of behavioral response in sports live streaming.

**Discussion:**

These findings suggest that viewer engagement in sports live streaming is shaped by both cognitive and affective processes, although these processes operate differently. By clarifying the mediating role of flow experience and the conditional role of emotional bond, this study offers a more nuanced understanding of how viewers become psychologically involved and behaviorally responsive in live-streaming environments. It also provides useful insights for platforms, influencers, and sports marketers seeking to strengthen viewer connection and encourage more sustained interaction.

## 1 Introduction

Sports live streaming has developed into an important part of today’s digital media environment, particularly on social media platforms where viewing is rarely a purely passive activity. Viewers do not simply watch what is happening; they react to it immediately, interact with others, and become involved in the unfolding event in real time. While live streaming has been examined in a variety of settings, sports live streaming deserves to be treated separately because it brings together several features that are especially powerful when combined: real-time consumption, uncertain outcomes, shared emotional intensity, and identification with particular teams or athletes. As a result, viewer responses in this context are shaped by more than platform functions alone. They are also influenced by suspense, rivalry, and the meanings viewers attach to moments as the event unfolds. This makes sports live streaming a useful setting for exploring how media cues are converted into different forms of viewer engagement.

In this study, sports live streaming refers to sports content that is transmitted online in real time through digital platforms where audiences can engage with the stream as it unfolds or around the streaming process itself. The term is used here to distinguish this setting from traditional live broadcasting, which is typically linked to scheduled one-way transmission on television or radio and tends to provide fewer immediate opportunities for audience interaction. Even though the two formats may sometimes overlap, our interest is centered on sports live streaming as an interactive digital setting rather than on conventional broadcast delivery. In this study, a social media influencer (SMI) refers to a content creator or recognizable community figure whose presence is rooted in social platforms and who regularly participates in sports live streaming. This participation may take the form of hosting, co-hosting, commenting, or selecting and organizing stream-related content. In this study, such figures are important because viewers may respond to them on the basis of perceived expertise, credibility, or attractiveness. The category includes not only independent sports vloggers and streamer-commentators, but also other community figures whose influence has been built primarily through social platforms rather than through formal broadcaster roles alone.

Live-streaming research has already moved well beyond the exploratory stage. It is now a sizable multidisciplinary field, and existing review and overview studies have done a great deal to organize its key concepts, main research settings, and broader lines of development. One recurring point in these reviews is that game streaming, esports, and similar forms of platform-based spectatorship occupy a central place in the literature (Reitman et al., 2020; Cabeza-Ramírez et al., 2021; Woodcock and Johnson, 2019). Beyond these areas, studies in neighboring live-streaming contexts have examined audience participation, the role of platform affordances, motives for watching, social spending, immersion, and different kinds of relational dynamics in considerable detail (Taylor, 2018; Wohn and Freeman, 2019; Ma et al., 2021; Ye et al., 2023). Work on monetized interaction has likewise shown that flow, social presence, and interaction-related cues can influence behaviors such as virtual gifting, user support, and purchasing responses in social live streaming and live-streaming commerce (Li et al., 2018, 2024; Chang and Yu, 2023; Tang et al., 2024; Wang et al., 2025). This literature indicates that live streaming is already a well-developed domain of inquiry with strong psychological, sociocultural, and platform-based foundations.

However, findings from these better-developed subfields cannot be assumed to apply directly to sports live streaming. Relative to game streaming, esports, gifting-based streaming, or live-streaming commerce, sports live streaming places viewers in a setting where unfolding competition, shifting momentum, and shared fan identification may reorganize how influencer cues are perceived and acted upon. In such a setting, influencers do more than present content. They may interpret fast-moving events, frame competitive meaning, intensify emotional reactions, and connect viewers through shared fandom and commentary. For this reason, sports live streaming provides a useful context for examining whether established psychological mechanisms in adjacent live-streaming fields operate in similar or differentiated ways when the content is tied to real-time competition and collective affective involvement. More importantly, this context may alter how immersive experience is translated into downstream behavioral expression. Because sports live streaming is shaped by real-time competition, collective affect, and identity-laden spectatorship, immediate in-stream participation and outward content sharing may become more weakly coupled than in other live-streaming contexts.

This distinction is particularly important for understanding viewer engagement. In this study, viewer engagement refers to non-monetary behavioral responses that occur during the live stream or extend beyond it. It does not include influencers’ own broadcasting behavior or transaction-based actions such as tipping, gifting, paid subscriptions, or direct purchases. Interactivity, in turn, refers to platform-enabled viewer actions such as commenting, liking, reacting, and sharing content during or around the stream, again excluding monetary behaviors. More specifically, this study also differentiates between participative behavior and sharing behavior. The former refers to what viewers do directly during the stream, such as liking, commenting, reacting, or otherwise taking part in the live session. The latter concerns whether viewers pass stream-related content on to others by sharing, recommending, or forwarding it within their social network. While the two are both forms of engagement, they should not be treated as identical. Participating in a stream may require little more than immediate involvement, whereas sharing often carries greater social visibility and may depend more on identity alignment, perceived social value, or self-presentation concerns.

Against this background, some important questions in sports live streaming remain unresolved. One is how influencer characteristics and flow experience can be understood together in this setting, even though both have already received considerable attention in related live-streaming research. Another concerns emotional bond, which has usually been viewed as something that develops through repeated interaction, rather than as a factor that may shape how influencer-related cues turn into immersion. A further issue involves viewer engagement. This is often treated as a single broad outcome, even though participative behavior and sharing behavior may operate as different response patterns in sports live streaming rather than as consecutive stages. These unresolved issues form the basis of the three research questions examined in this study.

RQ1: How do SMI characteristics shape viewers’ flow experience in sports live streaming?

RQ2: How does emotional bond condition the effects of social media influencer characteristics on viewers’ flow experience in sports live streaming?

RQ3: How is flow experience associated with participative behavior and sharing behavior, and should these two forms of engagement be understood as distinct outcomes rather than sequential ones?

To address these questions, this study draws on the Stimulus-Organism-Response framework and analyzes data from sports live streaming viewers in China. The remainder of the paper is organized as follows: section “2 Theoretical background and hypothesis formulation” presents the theoretical framework and research hypotheses; section “3 Methodology” describes questionnaire design and data collection; section “4 Analysis results and hypothesis testing” outlines the empirical results and hypothesis testing; section “5 Discussion” discusses theoretical and managerial implications; and section “6 Conclusion” offers concluding remarks and future research directions.

## 2 Theoretical background and hypothesis formulation

Sports live streaming is an interactive media context in which viewers respond not only to platform features and unfolding sports events, but also to cues conveyed by social media influencers (SMIs) who host, interpret, comment on, or curate the stream. To account for how these cues are translated into viewer responses, this study adopts the Stimulus-Organism-Response (SOR) framework. Within this framework, SMI characteristics are treated as external stimuli, flow experience represents the viewer’s internal psychological state, and viewer engagement serves as the behavioral response. In the present study, engagement is defined in non-monetary terms and focuses specifically on participative behavior and sharing behavior, excluding transactional actions such as tipping, gifting, paid subscriptions, and direct purchases. Emotional bond is further incorporated as a first-stage moderator, as it is expected to shape the extent to which influencer-related cues give rise to flow experience rather than the subsequent links from flow experience to behavioral outcomes. The SOR framework is particularly appropriate in this context because it provides a clear basis for explaining how communicator attributes, psychological immersion, and differentiated forms of engagement are connected in sports live streaming.

### 2.1 SMI characteristics (expertise, credibility and attractiveness)

Prior research suggests that communicator characteristics play an important role in shaping how audiences evaluate messages, place trust in information, and develop behavioral responses (Hovland and Weiss, 1951; Lafferty and Goldsmith, 1999; Erdogan, 1999). Their role may be even more consequential in live-streaming environments, where viewers encounter influencer cues in real time and respond under conditions of immediacy, emotional intensity, and continuous interaction. Within sports live streaming, three characteristics are especially relevant for understanding these processes: expertise, credibility, and attractiveness.

Expertise reflects the extent to which an SMI is perceived as knowledgeable and capable of providing accurate and useful information (Bansal and Voyer, 2000). In sports live streaming, this quality goes beyond general product or topic knowledge and includes the ability to explain rules, interpret tactics, comment on performance, and make sense of rapidly changing match situations. When viewers regard an influencer as knowledgeable, they are more likely to rely on that person’s interpretations, process information more efficiently, and remain cognitively involved in the live-streaming experience. Prior research indicates that expertise can enhance persuasive influence while reducing the effort required to evaluate communicator-provided information (Till and Busler, 2000; Mattke et al., 2020). In interactive sports live streaming, these qualities are likely to foster sustained attention and immersion.

Credibility concerns whether an SMI and the information provided are seen as trustworthy, reliable, and believable (Dwivedi et al., 2018; AlFarraj et al., 2021). In sports live streaming, this quality is particularly important because viewers are continuously exposed to real-time commentary, interpretation, and affective framing. When an influencer is regarded as credible, viewers are more likely to see that person as sincere, consistent, and dependable, which can reduce uncertainty and support continued psychological involvement in the stream. Prior research indicates that communicator credibility is central to the development of audience trust, persuasive influence, and behavioral intention formation (Schouten et al., 2020; Belanche et al., 2021). In interactive sports live streaming, these effects are likely to help sustain attentional focus and reinforce immersion in unfolding content.

Attractiveness involves more than physical appeal. It also encompasses charisma, expressive style, screen presence, and other socially appealing personal qualities (Erdogan, 1999; Horai et al., 1974). In sports live streaming, this dimension may also include athletic appeal, communicative energy, and a style of presentation that makes the stream feel more vivid and emotionally compelling. Influencers perceived as attractive are more likely to capture viewers’ attention, evoke favorable affect, and create an engaging viewing atmosphere. In immersive media settings, such qualities are likely to strengthen viewers’ sense of involvement and increase the likelihood that they become absorbed in the stream (Zheng, 2023).

Overall, expertise, credibility, and attractiveness can be understood as key SMI-related cues that shape how viewers perceive and experience sports live streams. When these characteristics are positively evaluated, the stream is more likely to be experienced as engaging, coherent, and absorbing, which may in turn strengthen viewers’ flow experience. On this basis, the following hypotheses are developed:

**H1: SMI characteristics positively influence viewers’ flow experience.**

**H1-1: SMI expertise positively influences flow experience.**

**H1-2: SMI credibility positively influences flow experience.**

**H1-3: SMI attractiveness positively influences flow experience.**

### 2.2 Flow experience

Csikszentmihalyi (1988) described flow experience as a state of deep absorption in which individuals become intensely focused on an ongoing activity and experience a strong sense of involvement together with intrinsic enjoyment. In interactive media research, flow has become an important concept for explaining how external cues contribute to sustained attention, immersion, and subsequent behavioral responses.

Research on related live-streaming contexts has already shown that flow and immersion play an important psychological role. Flow has been found to mediate virtual gift purchasing in live streaming (Li et al., 2018), while later studies have linked gifting behavior to broader motivational structures in social live-streaming services (Tang et al., 2024). In live-streaming commerce, gamification, presence, and immersion have likewise been associated with purchase intention and related behavioral outcomes, and interaction-immersion models suggest that streamer attractiveness can strengthen immersion-related effects (Chang and Yu, 2023; Li et al., 2024). Together, these findings indicate that flow is already a well-established organismic mechanism in live-streaming research. What remains less clearly specified is how flow shapes non-transactional viewer engagement in sports live streaming, rather than monetized participation, gifting, or commerce-related behavior.

In this study, flow is treated not simply as a positive subjective reaction, but as an intermediate psychological state through which viewers’ perceptions of SMI characteristics are translated into behavioral engagement. In sports live streaming, audiences encounter fast-moving events, emotionally charged turning points, and continuous influencer commentary in real time (Dong et al., 2023; Ock et al., 2024). Within this context, flow represents a meaningful state of concentration and immersion that connects influencer-related cues with later responses. This interpretation fits the SOR framework, in which organismic states serve to mediate the effects of external stimuli on behavior.

The behavioral responses associated with flow should not be treated as uniform. In this study, viewer engagement is differentiated into participative behavior and sharing behavior. Participative behavior includes immediate, platform-based actions such as liking, commenting, reacting, or otherwise taking part in the live-streaming session (Giertz et al., 2022; Lv et al., 2022). These actions are typically synchronous, relatively low in behavioral threshold, and closely connected to the unfolding viewing experience. By contrast, sharing behavior involves recommending, forwarding, or redistributing stream-related content to others through a broader social network (Shi et al., 2014; Ham et al., 2019). Relative to participative behavior, sharing is more publicly visible and more likely to involve self-presentational concerns, evaluations of social value, and expectations about audience feedback.

This distinction is particularly important in sports live streaming, where viewers are often highly immersed and emotionally activated during the viewing process (Rejikumar et al., 2022). In such conditions, flow may encourage both immediate participation within the stream and outward sharing beyond it (Carlson et al., 2017). At the same time, these two forms of engagement differ in their action context and psychological basis. Participative behavior is embedded in the live session itself, whereas sharing behavior extends into broader networked self-presentation (Swani and Labrecque, 2020). They may therefore be related without necessarily constituting a sequential process. Even so, prior research on engagement often suggests that lower-threshold forms of participation can develop into more outward and socially expressive responses (Gavilanes et al., 2018; Aldous et al., 2019; Kumar et al., 2022). In live-streaming environments, viewers who participate actively may become more psychologically involved in the stream and more likely to extend that involvement through subsequent sharing. From this perspective, the following hypotheses are advanced:

H2: Flow experience positively influences viewer engagement during sports live streaming.

H2-1: Flow experience positively affects participative behavior.

H2-2: Flow experience positively affects sharing behavior.

H3: Participative behavior positively influences sharing behavior.

In addition to these direct effects, flow is expected to operate as an intervening mechanism linking SMI characteristics to viewer engagement (Li et al., 2018; Zhang et al., 2025; Wang and Cai, 2025). When influencers are perceived as knowledgeable, credible, and attractive, viewers are more likely to become attentive, absorbed, and immersed in the live-streaming experience. This heightened state of flow may, in turn, increase the likelihood of both participative behavior and sharing behavior. The following hypotheses are proposed:

H4: Flow experience mediates the relationship between SMI characteristics and participative behavior.

H4-1: Flow mediates the effect of expertise on participative behavior.

H4-2: Flow mediates the effect of credibility on participative behavior.

H4-3: Flow mediates the effect of attractiveness on participative behavior.

H5: Flow experience mediates the relationship between SMI characteristics and sharing behavior.

H5-1: Flow mediates the effect of expertise on sharing behavior.

H5-2: Flow mediates the effect of credibility on sharing behavior.

H5-3: Flow mediates the effect of attractiveness on sharing behavior.

### 2.3 Emotional bond

As social media has become more embedded in everyday communication, viewers have increasingly developed relationship-like ties with influencers through repeated exposure, ongoing interaction, and mediated familiarity. Research on parasocial relationships indicates that audiences may experience intimacy, attachment, and psychological closeness toward media figures even when communication remains largely one-sided (Alperstein, 2019). In platform-based environments, these connections can be further strengthened by repeated commenting, replies, live interaction, and the perceived accessibility of influencers, which may foster stronger emotional bonds between viewers and content creators (Chambers, 2013; Choi and Lee, 2019).

In sports live streaming, emotional bond may be particularly important because influencers do more than simply provide content. They interpret fast-changing events, express emotional reactions, perform fandom, and help viewers make sense of competition in socially meaningful ways. Through repeated co-viewing, commentary, and interaction, viewers may gradually develop a sense of psychological connection with the influencer (Deng et al., 2022; Kim et al., 2022). This relational closeness may then shape how influencer-related cues are interpreted in later live-streaming sessions. From this perspective, emotional bond is not only a product of prior exposure, but also a relational condition that may influence subsequent psychological responses.

Earlier research has often regarded emotional bond, or related parasocial constructs, as outcomes of repeated media exposure and interaction, typically reflected in fan attachment, loyalty, or relational closeness (Cohen, 2004; Eyal and Dailey, 2012; Ma et al., 2021). The present study adopts a different perspective. Rather than treating emotional bond as a downstream consequence of engagement, it is positioned here as a first-stage moderator within the SOR framework. More specifically, emotional bond is expected to shape how SMI characteristics influence flow experience, rather than how flow experience subsequently affects participative behavior or sharing behavior. This conceptualization is theoretically appropriate because emotional bond operates at the stage where communicator-related cues are psychologically received and interpreted. Before viewers decide whether to participate or share, they first encounter the influencer as a meaningful social presence. When viewers feel emotionally connected to an influencer, they may be more receptive to that SMI’s expertise, more responsive to that SMI’s credibility, and more likely to experience the presentation style as engaging and immersive (Lim and Lee, 2023; Ohlin and Gyllén, 2025; Nguyen and Hoang, 2026). Emotional bond may therefore strengthen the extent to which influencer-related cues are internalized as flow experience.

This first-stage moderating role may be especially important in sports live streaming. Because sports content unfolds in real time and is often accompanied by intense emotion, suspense, and shared attention, viewers may depend more heavily on trusted and emotionally resonant influencers when interpreting and experiencing the stream. In this context, emotional bond may strengthen the effects of expertise, credibility, and attractiveness on viewers’ immersion. The following hypotheses are accordingly proposed:

**H6: Emotional bond moderates the relationship between SMI characteristics and flow experience.**

**H6-1: Emotional bond moderates the effect of expertise on flow experience.**

**H6-2: Emotional bond moderates the effect of credibility on flow experience.**

**H6-3: Emotional bond moderates the effect of attractiveness on flow experience.**

### 2.4 Research model

**FIGURE 1:**
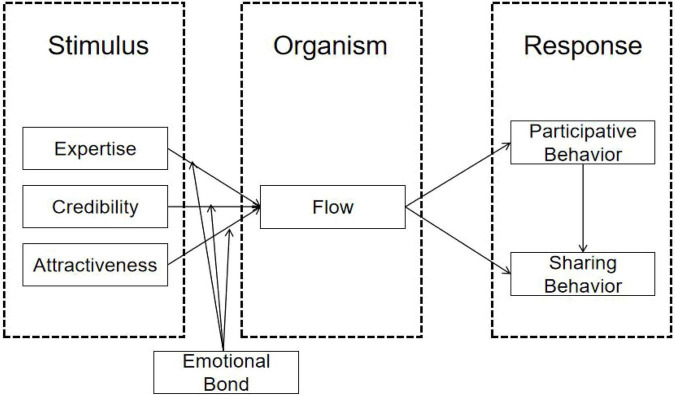
Research model.

The proposed model is developed on the basis of the SOR framework. In this model, SMI expertise, credibility, and attractiveness function as external stimuli, while flow experience represents the organismic state linking these cues to psychological immersion. Participative behavior and sharing behavior are treated as two distinct behavioral responses rather than as a single form of engagement. Emotional bond is positioned as a first-stage moderator, shaping the effects of SMI characteristics on flow experience. Figure 1 presents the study’s conceptual model.

## 3 Methodology

### 3.1 Questionnaire design

The items used to measure each construct in this study were adapted from previous studies and included SMI characteristics (expertise, credibility, and attractiveness), flow experience, participative behavior, sharing behavior, and emotional bond. This study used a five-point Likert scale, with 1 indicating strongly disagree and 5 indicating strongly agree. Most scale items were initially developed in English, the questionnaire was translated into Chinese after expert review and revision before being distributed to respondents.

Social media influencer characteristics were measured using the scale developed by Choi and Lee (2019), while flow experience was assessed with the scale reported by ShiYong et al. (2023). In this study, viewer engagement was operationalized through two distinct dimensions: participative behavior and sharing behavior. Participative behavior captures viewers’ immediate, platform-based engagement during the live-streaming session, such as liking, commenting, or reacting in real time. Sharing behavior, by contrast, reflects viewers’ tendency to redistribute, repost, forward, or recommend live-stream content to others through their broader social network. Although both dimensions were adapted from Zheng (2023), they were modeled as separate latent variables because they represent different forms of non-transactional engagement. Participative behavior was measured with three items (PB1–PB3), and sharing behavior was measured with three retained items (SB2–SB4), following item refinement during the pilot stage. The wording of these items was slightly revised to better fit the sports live-streaming context while preserving their original conceptual meaning.

Because this study relied on a standardized survey design, the questionnaire focused on respondents’ general perceptions and self-reported behavioral tendencies rather than on event-specific narratives or detailed accounts tied to particular streams. The resulting data therefore capture broader patterns in how viewers perceive influencer characteristics, experience flow, and report engagement tendencies across sports live-streaming contexts. Analyses of specific participation or sharing episodes at the case level are beyond the scope of the present study.

To minimize the risk of common method bias, several procedural steps were taken during the design and administration of the questionnaire. Respondents were informed that the survey was anonymous and that there were no right or wrong answers, which helped reduce evaluation apprehension and socially desirable responding. In addition, all constructs were measured with previously validated scales that were reviewed and refined before the formal survey was launched. The wording of the questionnaire was also adjusted to improve clarity and reduce ambiguity in how respondents interpreted the items. Together, these procedures were intended to enhance response quality and limit method-related bias at the design stage.

### 3.2 Data collection and sample description

This study used a cross-sectional survey with convenience sampling. To qualify for participation, respondents needed prior experience watching influencer-hosted sports live streams on interactive digital platforms. To ensure that participants understood the research context consistently, brief definitions of the focal terms were provided before the main questionnaire. “Sports live streaming” was described as real-time sports content delivered online through digital platforms that allow synchronous viewer interaction. “Social media influencer” referred to a follower-based sports content creator, streamer, or community figure who regularly hosts, co-hosts, or comments on sports live streams. Participants were instructed not to base their responses on traditional television or radio broadcasters or on official institutional sports channels, unless those figures were primarily followed as social-media personalities on interactive platforms. They were also instructed to answer the questionnaire with the influencer-hosted sports live stream they watched most often in mind.

Before entering the main survey, participants first answered a screening question asking whether they had prior experience watching influencer-hosted sports live streams. Only those who met this requirement were allowed to continue. To reach potential users of sports live streaming, the Wenjuanxing survey link and QR code were circulated through online channels likely to include relevant audiences, such as WeChat contacts and group chats, social-media reposting, and online communities related to sports viewing and live-streaming use. As the study focused on sports live-streaming viewers in China, recruitment was carried out in a context closely connected to mainstream platforms such as Douyin, Kuaishou, and Bilibili, although participation was not limited to any single platform or sport category.

A pilot test involving 50 participants was carried out to assess the questionnaire before the formal survey. Based on the feedback obtained at this stage, items that were unclear or redundant were revised or removed. The final questionnaire was distributed through the Chinese online survey platform Wenjuanxing^1^ between 1 December and 20 December 2024. A total of 650 responses were received, and 617 were retained as valid after 33 incomplete or inconsistent submissions had been excluded.

The demographic profile of the respondents indicated a relatively balanced gender distribution, with 52.5% male and 47.5% female participants. In terms of age, 52.4% were younger than 30, and the largest subgroup (28.8%) fell within the 21–30 age range. This pattern is broadly consistent with the core user base of major sports live-streaming platforms in China, such as Douyin, Kuaishou, and Bilibili, suggesting that the sample was drawn from the mainstream sports live-streaming ecosystem. With respect to income, 61.5% of respondents reported a monthly income below RMB 5,000. In terms of occupation, the sample was composed mainly of students (27.4%), corporate employees (15.9%), and public-sector workers (16.0%).

Overall, the sample provides a suitable basis for testing the proposed model among Chinese viewers who have prior experience with sports live streaming. Still, the findings should not be read as nationally representative, since the study used convenience sampling and relied on self-reported survey data. The survey also did not restrict respondents to one particular sport category or streaming format. As a result, the findings are more appropriately interpreted as capturing a broad sports live-streaming context rather than any single sport or subgenre.

## 4 Analysis results and hypothesis testing

### 4.1 Test of normality

The data were analyzed using structural equation modeling in AMOS 25. Measurement and structural models were developed with maximum likelihood (ML) estimation, and we verified that the data satisfied ML’s normality assumptions. The results showed skewness ranging from **−**0.609 to 0.085 and kurtosis from **−**0.953 to -0.486, all within acceptable limits (| skew| < 3, | kurtosis| < 7), indicating the sample was approximately normal (Kline, 2015).

### 4.2 Measurement model assessment

#### 4.2.1 Common method bias assessment

Because the data were collected through self-reported questionnaires, common method bias was assessed with Harman’s single-factor test. Multiple factors showed eigenvalues greater than 1, and the first unrotated factor explained 33.43% of the total variance. This value was below the commonly used 50% threshold, suggesting that common method bias was not a serious issue in the study.

#### 4.2.2 Reliability and convergent validity

Cronbach’s alpha was used to assess internal consistency, with a value above 0.7 considered acceptable (Bagozzi and Yi, 1988). Cronbach’s alpha values for all constructs ranged from 0.750 to 0.824, exceeding the 0.7 threshold and indicating adequate reliability.

Hair et al. (2014) suggested that models should be evaluated for convergent and discriminant validity. Convergent validity was assessed via CR and AVE values for each construct. As shown in Table 1, CR values for all constructs ranged from 0.751 and 0.830, which met the criterion of 0.7, and AVE values for all constructs ranged from 0.502 and 0.624, which also met the criterion of 0.5 (Fornell and Larcker, 1981). Additionally, Hair et al. (2011) noted that factor loadings of each item should be exceed 0.6 to establish convergent validity, in our model all loadings fell between 0.656 and 0.920, satisfying this condition. Therefore, the measurement model exhibits acceptable convergent validity.

**TABLE 1:** Results of confirmatory factor loadings, reliability, and convergent validity.

Full name	Items	UNSTD	S.E.	*t*-value	STD	Cronbach’s α	CR	AVE
Sharing behavior	SB3	1.000	–	–	0.745	0.772	0.773	0.531
	SB2	0.956	0.070	13.602***	0.725			
	SB4	0.934	0.069	13.583***	0.716			
Participative behavior	PB1	1.000	0.043	15.214***	0.878	0.804	0.808	0.587
	PB2	0.650			0.703	–	–	–
	PB3	0.654	0.043	15.236***	0.705	–	–	–
Expertise	Exp3	1.000	–	–	0.742	0.750	0.751	0.502
	Exp4	0.985	0.079	12.397***	0.724	–	–	–
	Exp5	0.897	0.073	12.280***	0.656	–	–	–
Attractiveness	Attr4	1.000	–	–	0.744	0.763	0.763	0.518
	Attr2	0.903	0.069	13.080***	0.708	–	–	–
	Attr3	0.938	0.072	13.077***	0.707	–	–	–
Flow experience	FE2	1.000	–	–	0.772	0.800	0.800	0.571
	FE3	0.959	0.063	15.288***	0.742	–	–	–
	FE4	0.967	0.063	15.339***	0.753	–	–	–
Credibility	Cred4	1.000	–	–	0.739	0.771	0.771	0.529
	Cred2	0.818	0.061	13.513***	0.721	–	–	–
	Cred5	0.994	0.074	13.514***	0.721	–	–	–
Emotional bond	EB1	1.000	–	–	0.920	0.824	0.830	0.624
	EB4	0.676	0.040	16.869***	0.728	–	–	–
	EB5	0.654	0.040	16.450***	0.703	–	–	–

*p < 0.05, **p < 0.01, ***p < 0.001.

#### 4.2.3 Discriminant validity

Discriminant validity was examined using the heterotrait-monotrait ratio (HTMT), which is often regarded as more sensitive than the traditional Fornell-Larcker criterion (Ab Hamid et al., 2017). Under the HTMT criterion, values below 0.85 indicate an acceptable level of discriminant validity. As reported in Table 2, all HTMT values in this study fell between 0.344 and 0.578, supporting adequate discriminant validity across the constructs. In particular, the HTMT value between participative behavior and sharing behavior was 0.344, well below the recommended cutoff, which further supports the empirical distinction between these two forms of engagement.

**TABLE 2:** Discriminant validity [heterotrait-monotrait (HTMT) criteria].

	(1)	(2)	(3)	(4)	(5)	(6)
Expertise (1)	–	–	–	–	–	–
Credibility (2)	0.465	–	–	–	–	–
Attractiveness (3)	0.46	0.555	–	–	–	–
Flow experience (4)	0.473	0.472	0.56	–	–	–
Participative behavior (5)	0.387	0.392	0.457	0.415	–	–
Sharing behavior (6)	0.477	0.503	0.578	0.475	0.344	–

### 4.3 Direct effects

The model fit indices indicated a good fit: χ2 = 248.819, χ2/df = 1.975 (<3), RMR = 0.077 (<0.1), and RMSEA = 0.0622 (<0.08), which met the criteria. Additionally, GFI = 0.958, AGFI = 0.943, NFI = 0.937, RFI = 0.924, IFI = 0.968, TLI = 0.961, and CFI = 0.968, all above the 0.9 benchmark. Overall, the theoretical model’s fit was acceptable.

Table 3 reports the direct relationships among the focal constructs. Expertise showed a significant positive association with flow experience (H1-1: β = 0.274, *t* = 4.475, *p* < 0.001), and similar positive effects were found for credibility (H1-2: β = 0.195, *t* = 3.163, *p* < 0.01) and attractiveness (H1-3: β = 0.421, *t* = 6.494, *p* < 0.001). Flow experience was also positively related to both participative behavior (H2-1: β = 0.602, *t* = 9.086, *p* < 0.001) and sharing behavior (H2-2: β = 0.525, *t* = 8.576, *p* < 0.001). By contrast, the path from participative behavior to sharing behavior was not significant (H3: β = 0.078, *t* = 1.893, *p* = 0.058). These results indicate that flow experience directly encouraged both forms of engagement, whereas participative behavior did not show a significant direct effect on sharing behavior in the present model.

**TABLE 3:** Standardized structural estimates.

Hypothesis path		Standardized estimates	Standardized error	*t*-value	Result
H1-1	Expertise→flow experience	0.274	0.061	4.475***	Support
H1-2	Credibility→flow experience	0.195	0.062	3.163**	Support
H1-3	Attractiveness→flow experience	0.421	0.065	6.494***	Support
H2-1	Flow experience→participative behavior	0.602	0.066	9.086***	Support
H2-2	Flow experience→sharing behavior	0.525	0.061	8.576***	Support
H3	Participative behavior→sharing behavior	0.078	0.041	1.893	Reject

**p < 0.01, ***p < 0.001.

### 4.4 Indirect effects of flow experience

To further examine the mediating role of flow experience, indirect effects were tested using a phantom model approach with bootstrapped confidence intervals. As reported in Table 4, all six indirect paths from SMI characteristics to the two engagement outcomes through flow experience were significant. For participative behavior, flow experience significantly mediated the effects of expertise (indirect effect = 0.165, 95% CI [0.084, 0.259], *p* = 0.001), credibility (indirect effect = 0.118, 95% CI [0.034, 0.200], *p* = 0.009), and attractiveness (indirect effect = 0.253, 95% CI [0.165, 0.363], *p* = 0.001). The same pattern was observed for sharing behavior, with significant indirect effects for expertise (indirect effect = 0.144, 95% CI [0.073, 0.234], *p* = 0.001), credibility (indirect effect = 0.102, 95% CI [0.031, 0.182], *p* = 0.009), and attractiveness (indirect effect = 0.221, 95% CI [0.132, 0.319], *p* = 0.001).

**TABLE 4:** Mediating effects.

Mediating path		Indirect effect	95% bootstrapping confidence intervals	*P*-value
H4-1	Expertise→flow experience→participative behavior	0.165	0.084∼0.259	0.001
H4-2	Credibility→flow experience→participative behavior	0.118	0.034∼0.200	0.009
H4-3	Attractiveness→flow experience→participative behavior	0.253	0.165∼0.363	0.001
H5-1	Expertise→flow experience→sharing behavior	0.144	0.073∼0.234	0.001
H5-2	Credibility→flow experience→sharing behavior	0.102	0.031∼0.182	0.009
H5-3	Attractiveness→flow experience→sharing behavior	0.221	0.132∼0.319	0.001

None of the bootstrapped confidence intervals included zero, providing further evidence that flow experience significantly mediated the relationships between SMI characteristics and both participative behavior and sharing behavior.

### 4.5 Moderating effects of emotional bond

To examine the moderating role of emotional bond, a multi-group SEM analysis was performed by comparing constrained and unconstrained models across low and high emotional bond groups. In the present model, emotional bond was specified as a first-stage moderator affecting the links between SMI characteristics and flow experience.

As reported in Table 5, emotional bond significantly moderated the path from expertise to flow experience (Δx^2^ = 8.814, *p* = 0.003) as well as the path from credibility to flow experience (Δx^2^ = 5.716, *p* = 0.017). By contrast, its moderating effect on the path from attractiveness to flow experience was not significant (Δx^2^ = 2.965, *p* = 0.085). These results suggest that emotional bond does not uniformly strengthen all structural relationships in the model. Instead, its effect appears to be selective, reinforcing the influence of expertise and credibility on flow experience.

**TABLE 5:** Moderating effects.

Moderating path		△df	△χ 2	*P*-value
H6-1	Expertise→flow experience (moderating effect of emotional bond)	1	8.814	0.003
H6-2	Credibility→flow experience (moderating effect of emotional bond)	1	5.716	0.017
H6-3	Attractiveness→flow experience (moderating effect of emotional bond)	1	2.965	0.085

Additional group comparisons showed that the effects of expertise and credibility on flow experience were stronger in the high emotional bond group than in the low emotional bond group. No significant between-group difference was found for the effect of attractiveness on flow experience. This pattern indicates that emotional bond amplifies cognitively grounded influencer cues rather than all forms of SMI appeal.

## 5 Discussion

### 5.1 Findings

This study focused on viewer engagement in sports live streaming and, in particular, distinguished between two non-transactional forms of engagement: participative behavior and sharing behavior. The findings show that expertise, credibility, and attractiveness all contributed positively to flow experience, with attractiveness emerging as the strongest predictor. Flow experience, in turn, was positively associated with both participative behavior and sharing behavior. These results align with earlier research showing that immersive psychological states can encourage user involvement, exploratory action, and continued engagement in digital environments (Chen and Lin, 2018; Hyun et al., 2021; Zheng, 2023). Prior studies in related settings, such as social live streaming and live-streaming commerce, have likewise linked flow and immersion to gifting, purchase-related responses, and interaction intensity (Li et al., 2018, 2024; Chang and Yu, 2023; Tang et al., 2024). The present study extends this literature by showing that in sports live streaming, flow experience is connected not to a single undifferentiated form of engagement, but to multiple forms of viewer response.

One notable finding is that the direct path from participative behavior to sharing behavior was not significant. This suggests that the two should not be understood as a simple linear sequence. In sports live streaming, immediate in-stream participation does not necessarily develop into broader content diffusion. Instead, participative behavior and sharing behavior appear to reflect different response tendencies. Earlier studies have often treated liking, commenting, and sharing as closely related or cumulative expressions of online engagement (Choi and Lee, 2019; Kim and Johnson, 2016), but the present results indicate that this assumption may not transfer directly to sports live streaming. It is also important to note that this interpretation is based on aggregate survey patterns rather than event-level observation. The findings do not suggest that all viewers follow the same behavioral route; rather, they indicate that the overall response structure in this sample is better captured by differentiated engagement paths than by a single sequential process. In this respect, the study also adds to research on game streaming, esports spectatorship, and relational labor in live-streaming environments, where participation, spending, and social connection have often been examined together, but where the distinction between immediate in-stream interaction and outward content redistribution has received less direct attention (Taylor, 2018; Wohn and Freeman, 2019; Ma et al., 2021; Ye et al., 2023).

This divergence may be better understood in relation to the distinctive affective structure of sports live streaming. Unlike many other live-streaming genres, sports live streaming is shaped by uncertain competitive outcomes, team allegiance, rivalry, and collectively experienced turning points. Events such as a goal, a controversial referee decision, a comeback, or a missed chance can trigger immediate and synchronized emotional reactions among viewers. In this context, participative behavior is not simply a low-threshold response enabled by platform interactivity. It often functions as a form of real-time affective alignment with unfolding competition and shared spectatorship. Sharing behavior, by contrast, moves beyond the immediate co-viewing setting and enters a broader social space, where sporting loyalties, audience boundaries, and identity expression become more visible. A viewer may therefore be very willing to comment or react in the moment while remaining more cautious about redistributing the same content publicly. This may help explain why participative behavior and sharing behavior did not form a significant direct sequence in the present model.

The moderation results add an important layer to these findings. Emotional bond strengthened the effects of expertise and credibility on flow experience, but it did not significantly strengthen the effect of attractiveness. This indicates that relational closeness does not enhance every type of influencer appeal in the same way. Instead, viewers who feel emotionally connected to an influencer seem to respond more strongly to cognitively grounded cues such as knowledgeability and trustworthiness. In sports live streaming, this distinction is theoretically important because viewers often turn to influencers not only for entertainment, but also for interpretation, judgment, and emotional orientation as events unfold in real time.

### 5.2 Theoretical implications

The findings of this study have several theoretical implications. One theoretical contribution of this study lies in its extension of the Stimulus-Organism-Response (SOR) framework to the context of sports live streaming. More specifically, the findings identify flow experience as a central organismic mechanism through which SMI characteristics are linked to viewer engagement. Although flow has been widely examined in online shopping, gamification, and related live-streaming contexts, its role in shaping non-transactional engagement in sports live streaming has received less direct attention (Hoffman and Novak, 2009; Hamari et al., 2016; Wongkitrungrueng and Assarut, 2020). By showing that expertise, credibility, and attractiveness all contribute to flow experience, and that flow in turn predicts both participative behavior and sharing behavior, the study broadens the application of the SOR framework in digitally mediated sports spectatorship.

Another theoretical contribution concerns the conceptualization of viewer engagement. The study distinguishes participative behavior from sharing behavior and shows that the two are not only conceptually different, but also empirically separable response forms. Earlier research has often grouped commenting, liking, and sharing together under broad notions of online engagement (Brodie et al., 2013; Dessart et al., 2015; Dolan et al., 2016), yet the present findings suggest that such aggregation may conceal meaningful differences. In sports live streaming, immediate in-stream interaction and outward content diffusion appear to involve different thresholds of action, different levels of social visibility, and partly different motivational bases. By showing that participative behavior did not significantly predict sharing behavior, the study challenges the assumption that these two behaviors should be treated as automatically sequential stages within a single engagement continuum.

A further theoretical contribution of this study lies in how it repositions emotional bond within viewer-influencer relational dynamics. Rather than treating emotional bond simply as an outcome of engagement, the study conceptualizes it as a boundary condition that shapes how influencer-related cues are processed. Previous research has often viewed parasocial closeness and emotional attachment as consequences of repeated exposure or interaction (Cohen, 2004; Tukachinsky and Walter, 2020; Bond, 2021). The present findings suggest a different possibility: emotional bond may influence the process at an earlier stage by affecting how viewers interpret influencer-related cues. More specifically, emotional bond strengthened the effects of expertise and credibility on flow experience, but not the effect of attractiveness. This indicates that relational closeness may amplify cognitively grounded cues more readily than purely affective or stylistic appeal, offering a more differentiated understanding of viewer-influencer relationships in live-streaming contexts.

The findings also highlight the importance of contextual specificity. Sports live streaming should not be treated as just another interactive media genre. It is a form of mediated spectatorship shaped by suspense, allegiance, rivalry, and collective interpretation. These features are theoretically important because they intensify real-time affective participation while also making outward sharing more dependent on identity expression and the management of audience boundaries. What matters, then, is not only the interactive nature of sports live streaming, but also the particular social and emotional conditions in which that interactivity unfolds. In this respect, the findings suggest that influencer and engagement theories can be developed further by paying closer attention to content-specific media environments.

### 5.3 Practical implications

The findings also carry practical implications for sports platforms, content creators, and brands that rely on influencer-led live-streaming strategies.

For sports platforms, content creators, and brands, the selection and development of influencers should not rely solely on visibility or charisma. Although attractiveness showed the strongest effect on flow experience, expertise and credibility also played meaningful roles, and their influence became even stronger when viewers had developed emotional bonds with the influencer. This suggests that sports live-streaming strategies should value not only presentational appeal, but also an influencer’s ability to offer knowledgeable and credible interpretation. In sports-related live content, audiences appear to respond not just to who is engaging, but also to who is informative and trustworthy.

Engagement strategies also need to distinguish between participative behavior and sharing behavior rather than treating them as interchangeable outcomes. When the goal is to stimulate activity within the live-streaming session, platforms and creators need to focus on conditions that support real-time immersion and emotional expression. When the goal is broader content diffusion, however, greater attention should be paid to the social value of the content, its relevance to viewers’ identities, and its potential to be shared outwardly. The logic of increasing comments or reactions during a live stream is therefore not necessarily the same as the logic of encouraging viewers to redistribute content beyond the session itself.

Community building should also be treated as a strategic resource rather than as a by-product of viewer interaction. Because emotional bond selectively increased the effects of expertise and credibility on flow experience, relational closeness appears to enhance the persuasive and immersive value of high-quality influencer cues. This suggests that long-term audience cultivation, trust building, and sustained familiarity between viewers and influencers matter not only for expanding audience size, but also for improving the quality of engagement. For this reason, platforms and creators should invest in continuity, relational consistency, and recognizable interaction styles that help build stronger viewer attachment over time.

### 5.4 Limitations and future research

Several limitations of this study point to useful directions for future research. One concerns the use of cross-sectional self-report data. Although the structural model supports the hypothesized relationships, the research design does not allow strong causal claims to be made. Future studies could address this limitation by using longitudinal designs, repeated-measures approaches, or experimental methods to examine how viewer engagement develops over time and to test the temporal ordering of the proposed relationships more directly.

Another limitation relates to the absence of event-level or case-level behavioral evidence. Because the study relied on standardized survey measures, the findings capture broad response patterns across viewers rather than showing how participation and sharing unfold within particular live-streaming episodes. Future research could address this gap by combining survey methods with qualitative interviews, diary approaches, or observational analyses of specific streaming events, thereby providing richer evidence on how these two forms of engagement diverge in practice.

A further limitation concerns the fact that the sample was drawn exclusively from Chinese viewers. Although this focus provides valuable contextual depth, it also constrains the broader generalizability of the findings. Viewer responses to expertise, credibility, emotional bond, and sharing may differ across cultural settings and platform environments. Future comparative research across countries or media systems would therefore be useful for assessing how robust the proposed model remains in other contexts.

In addition, this study did not distinguish respondents by sport category, even though all participants were sports live-streaming viewers. Different forms of sports live content, such as football, basketball, fitness, or esports-related streaming, may involve different levels of emotional arousal, identity attachment, and interaction norms. Future research could examine whether the relationships identified in this study vary across specific sports or across different types of sports live-streaming content.

One final limitation involves the scope of the proposed model. Although the model focuses on three primary influencer characteristics and one relational moderator, and this parsimonious design helps maintain conceptual clarity, it necessarily leaves out other factors that may also shape engagement. These may include perceived authenticity, platform trust, viewer personality, and platform-specific affordances. Future research could extend the model by incorporating such variables and by examining how technological features and social context jointly influence differentiated engagement in sports live streaming.

## 6 Conclusion

This study investigated how SMI characteristics are linked to viewer engagement in sports live streaming within the Stimulus-Organism-Response framework, flow experience, and emotional bond. The results indicate that expertise, credibility, and attractiveness all contribute to flow experience, that flow experience is positively associated with both participative behavior and sharing behavior, and that emotional bond reinforces part of this process by strengthening the effects of expertise and credibility on flow experience.

A key conclusion of this study is that viewer engagement in sports live streaming should not be understood as a single, undifferentiated construct. Participative behavior and sharing behavior are better seen as two related but distinct forms of engagement. The findings indicate that immediate in-stream participation does not automatically lead to outward sharing, pointing to a more differentiated response structure than a simple sequential pathway would suggest. This distinction is particularly meaningful in sports live streaming, where uncertainty, rivalry, and fan identification can generate intense real-time collective reactions without necessarily prompting viewers to redistribute content beyond the immediate viewing community.

The study contributes to research on digital engagement by showing that viewer responses in sports live streaming are shaped by the combined influence of influencer cues, immersive psychological states, and viewer-influencer relational dynamics. It also indicates that platforms, creators, and brands should distinguish among different engagement objectives rather than assuming that all interactive responses operate in the same way. As sports live streaming continues to evolve as an interactive media form, a clearer understanding of these differentiated engagement mechanisms will remain important for both theory and practice.

## Data Availability

The raw data supporting the conclusions of this article will be made available by the authors, without undue reservation.

## Footnotes

1
https://www.wjx.cn/
